# Difficult Airway Management in a Patient With Post-burn Contracture Neck

**DOI:** 10.7759/cureus.30011

**Published:** 2022-10-06

**Authors:** Deeksha Mishra, Vivek Chakole, Priyanka Dev

**Affiliations:** 1 Department of Anaesthesiology, Jawaharlal Nehru Medical College, Datta Meghe Institute of Medical Sciences, Wardha, IND

**Keywords:** i-gel, fibreoptic, difficult airway, contracture, burns

## Abstract

During reconstructive interventions in patients presenting with severe post-burn mento-sternal scar contracture, securing the airway forms a critical part of management. Extreme contracture is more likely to develop in patients who have had thoracic burns with ascending involvement of the neck and mandibular region. When cervical hyperextension and elevation of the mandible are impeded, post-burn contracture of the neck might render endotracheal intubation difficult. The development of rigid scar tissue that distorts the laryngeal and mandibular anatomy, or the development of microstomia following scar tissue retraction in facial burns, may make alternative approaches to direct laryngoscopy challenging. In patients with healed neck burns, intubation difficulties should be anticipated, and equipment for aiding intubation should be kept ready. Furthermore, a surgeon must be present throughout anesthesia induction in case an emergency neck release is required. Although the role of awake fiberoptic intubation has been well established in the general population, it is yet to be assessed in patients with burns. In this report, we present a case of successfully managed post-burn contracture that was planned for awake fiberoptic intubation.

## Introduction

The incidence of an unexpected difficult airway is reportedly 5.8%, and that of cannot intubate condition is 0.3% followed by cannot intubate cannot ventilate (CICV) situation comprising 0.003% of cases, all of which could be substantial causes of anesthesia-related morbidity and mortality [1]. In reconstructive surgeries involving patients with severe burns, appropriate airway management forms the most critical part of managing the patient. Anatomical variations may be extensive in these patients, rendering intubation challenging. Because the ability to provide oxygen to the patient is directly associated with mortality and morbidity, the airway must be handled with special caution in these patients. In this case report, we delineate the role of inhalational, intravenous, and local anesthetic agents along with fiberoptic intubation and I-gel in successfully managing a patient with circumoral and neck contracture leading to a fixated flexion deformity.

## Case presentation

A 35-year-old woman presented to the hospital with post-burn contracture of the neck and microstomia as a result of boiling water spilling over her face, neck, hands, chest, and abdomen.

**Figure 1:**
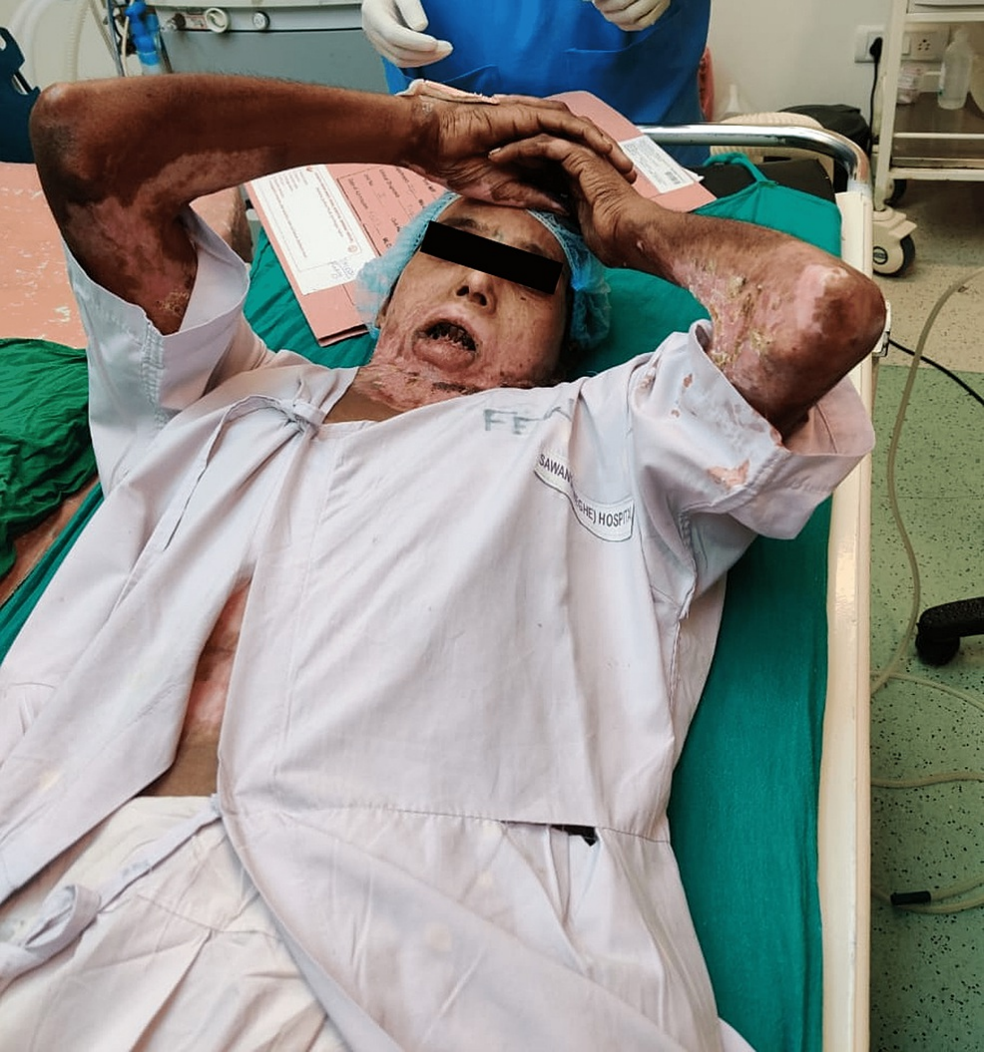
Flexion deformity of both upper limbs due to contracture

The patient was found to be American Society of Anaesthesiologists (ASA) grade one upon evaluation and had a contracture band spanning from her lower lip to around 10 cm below the base of the neck and one burn patch of approximately 10 x 7 cm on the abdomen extending till the umbilicus. The contracture had caused both of her upper limbs to flex, as depicted in Figure 1.

**Figure 2:**
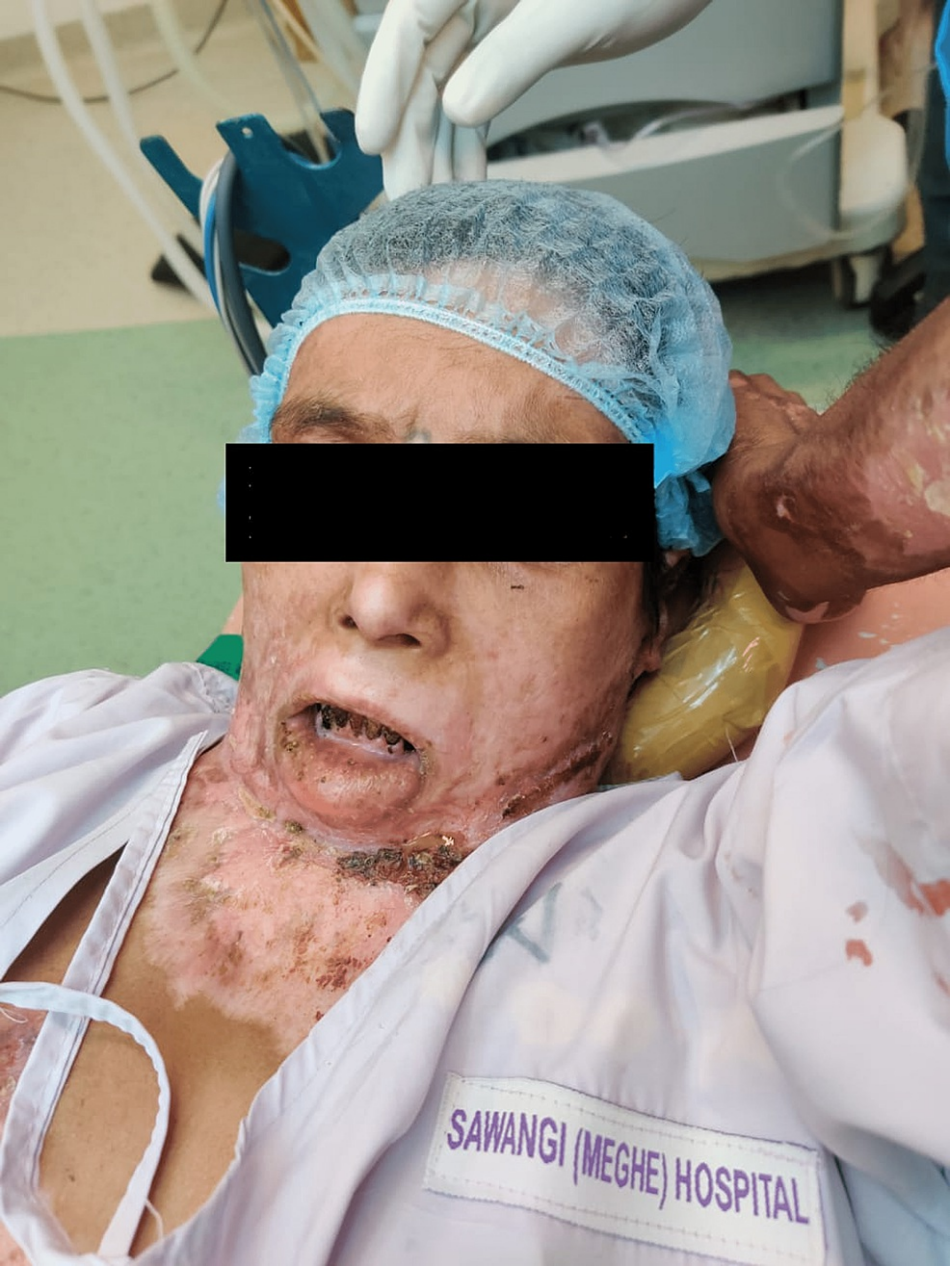
Fixed flexion deformity at the atlanto-occipital joint

As a result of the burn, the lower lip had become everted, the mucosa exposed, and the mouth opening inaccessible, resulting in microstomia. The contracture band on the neck had culminated in a fixed flexion deformity at the atlanto-occipital joint as represented in Figure 2, with no possibility of extension and the mouth opening reduced to one finger.

The X-ray of the head and neck revealed dental crowding, narrowed oropharyngeal spaces, decreased maxillo-pharyngeal angle, and reduced atlanto-occipital space. Upon evaluation, all laboratory tests were found to be within normal ranges and the systemic examination revealed no significant abnormalities. The patient was scheduled for a neck (mento-sternal) and circumoral contracture release procedure. Laryngeal mask airway (LMA), conventional laryngoscopy, or fiberoptic bronchoscopy (FOB) were ruled out due to the placement of the scar and the proposed surgery. As a result, the nasal route was chosen for performing awake fiberoptic intubation. The patient was counseled about the intubation procedure in detail with all the risks involved before obtaining due consent for the same, along with her approval to use her images for this case report.

Before taking the patient into the operating room, a 22-gauge intravenous cannula was secured in her right foot and the patient was nebulized with lidocaine 4% 45 minutes prior to the surgery, to anesthetize the upper airway with as minimal discomfort to the patient as possible. Usually, we employ a combination of techniques to deliver local anesthetic to the airway mucosa in preparation for awake fiberoptic intubation. But here the local nerve blocks could not be given due to the distorted anatomy of the neck due to contracture. Once inside the operating room, a femoral central line was secured for the patient as both the upper limbs had burn contractures and also taking into consideration the postoperative intensive care unit (ICU) care, which would be required for the patient. Standard monitoring commenced with attaching a five-lead electrocardiograph, blood pressure cuff (noninvasive monitoring), capnograph, and oxygen saturation probe. The surgeon was cautioned for a possible emergency tracheostomy, if needed, during the release of the contracture. The upper airway was anesthetized by instilling 1% lidocaine gel in the more patent (right) nostril before introducing a nasal airway in the same. The induction was started by pre-medicating the patient with intravenous glycopyrrolate 0.2 mg followed by pre-oxygenating for four minutes with oxygen flow at 6 liters per minute with a face mask kept closely over the mouth.

The flexible fiberoptic scope was introduced after the removal of the nasal airway from the right nostril. Our efforts were in vain because of the inadequate depth of local anesthesia provided that made the patient non-compliant upon introduction of the scope. After three unsuccessful attempts at intubating the patient, the release of labial commissures was planned as an emergency procedure after infiltration of 2% lidocaine with adrenaline as the local anesthetic agent following which the mouth opening was assessed again and found appropriate for the insertion of I-gel size 3. Intravenous midazolam 1 mg followed by 30 mg of ketamine and 30 milligrams of propofol were administered over five minutes in small doses while spontaneous respiration was ensured. Following intravenous induction, the plane of anesthesia was deepened by administering oxygen, nitrous oxide, and sevoflurane via I-gel to the spontaneously breathing patient. The intraoperative period was uneventful and I-gel was removed when the patient was awake and breathing spontaneously. The patient was then shifted to the surgical ICU for postoperative monitoring.

## Discussion

Patients with involvement of the face and neck resulting in scar contracture post-burn present a distinctive challenge when it comes to airway management. The underlying thick fibrous and hypertrophic scar sheets can deform the larynx and mandible. Due to severe mento-sternal contracture, the range of cervical motion may be restricted in all directions, making the sniffing position unfeasible. Posterior displacement of the mandible might be present with concomitant constraints in mobility. Facial burns in infancy can cause the jaw to underdevelop (micrognathia), resulting in further upper airway distortion [2]. Finally, a history of inhalation could indicate stenosis of the trachea, which could hinder the endotracheal tube from advancing further. Attempts at direct laryngoscopy may be unsuccessful as a result of these issues. Alternative methods of securing an airway should be considered only if conventional attempts at direct laryngoscopy prove to be in vain, according to the ASA Difficult Airway Algorithm [3].

Depending on the area of the burn or scar, the patient's physical parameters, the presence of any accompanying disease, and the skills and preferences of the operating surgeon, along with the availability of resources, these patients can be operated under both regional (local) and general anesthesia. Although a well-established method, tumescent anesthesia is not appropriate for raw areas greater than 10% and patients under the age of 18 years. Toxicity may result from the adverse effects of a significant dose of local anesthesia [4]. Choosing between awake intubation, local intervention, or maintaining spontaneous ventilation are three key concerns before inducing general anesthesia [5]. When the maxillo-pharyngeal angle is less than 90 degrees, the difficulty of direct laryngoscopy is equivalent to Cormack-Lehane classification grade III and IV, according to a prospective study of 148 individuals [6]. Furthermore, a reduction in the atlanto-occipital distance has long been recognized as a marker of difficult intubation. The difficulty in laryngoscopy is predicted using lateral cervical radiographic evaluation.

The sitting position is optimal for the assessment of the airway. The anesthesiologist should inspect the scar along with the contracture in addition to the regular assessment, scrutinizing the circumoral and perinasal region, as well as the size of the oral and nasal orifices. The mouth opening and cervical range of motion may be limited by mento-sternal contracture [7]. Burn scars on the oro-maxillofacial region might chaperone skeletal abnormalities that result in a small/receding jaw. The intubation route selected may be determined by the orchestration and formation of scar patterns. Finally, the epiglottis and the vocal cords might be pulled over to the direction of the scar and positioned anteriorly. If a laryngoscope is utilized, it should be advanced ipsilaterally towards the scar's direction.

If muscle relaxants are administered, the resilience of the scar tissue and the loss of pull by the adjacent tissues would exacerbate the retraction of the scar, rendering preoperative airway examination moot. The restricted mouth opening can become so profound that neither the laryngoscope blade nor the oral airway can pass through it. As a potential alternative route, the nasal orifices may also close down, making it impossible to insert a nasal airway.

Every intubation approach has its advantages and disadvantages. The laryngeal mask airway, for example, has been demonstrated to be a laudable airway adjunct for burn patients [8]. The external anatomical defects, recurring intraoperative position adjustments, and topical treatment to the parched area render it susceptible to displacement. The blind nasal method of intubation would be another option, but it requires the tongue to be anchored frontward by the contracture. This has its own set of drawbacks, namely limited positioning of the head and neck and the risk of nasal bleeding, which can make the provision of any supplemental instrumental aid entirely uncertain. If the airway anatomy is too distorted due to the retraction caused by soft tissue, bronchoscopy may be problematic [9]. Furthermore, repeated attempts may result in bleeding and discharge into the oropharynx. FOB has long been the touchstone for challenging intubations, with the added benefit of being pliable and navigable, enabling continuous structural visualization and a high rate of success [10], lowering the risk of trauma due to intubation and postoperative edema of the upper airway in patients with expected intubation difficulties. Sedation/total intravenous anesthesia or general anesthesia might be required in those patients for whom awake FOB is not possible or who are not willing to endure awake fiberoptic intubation. While inhalational anesthesia offers the advantage of maintaining spontaneous respiration, there is a danger of hypoventilation, upper airway obstruction, and hypoxemia while variations in the profundity of anesthesia and related cardiovascular and respiratory effects occur steadily.

## Conclusions

The keys to success in managing these patients are vigilance and planning. Sagacious preoperative airway and scar evaluation is essential in treating post-burn patients. Muscle relaxants should be avoided and spontaneous respiration should be maintained throughout. To manage the airway, the anesthesiologist should have a multi-layered contingency strategy in place. Because direct laryngoscopy has a strong probability of failure in these patients, indirect laryngoscopy should be attempted first. All intubation approaches in patients with significant deformity may fail due to underlying functional and anatomical changes. A surgeon should be on standby and prepared to step in if there is a need for a surgical release or an emergency tracheotomy. The ventilation, not the intubation, is what saves the patient's life.
